# Morphological & chemo mechanical analysis of exposed cervical dentin treated with three different desensitizing pastes (comparative in vitro study)^⋆^

**DOI:** 10.1016/j.jobcr.2025.06.018

**Published:** 2025-07-07

**Authors:** Manar S. Mohammad, Rasha H. Jehad

**Affiliations:** Department of Restorative and Aesthetic Dentistry, College of Dentistry, University of Baghdad, Iraq

**Keywords:** Theobromine, Nano-HAP, BAG, Dentin hypersensitivity, Raman microscopy and microhardness

## Abstract

**Background:**

Obstructing dentinal tubules is a valuable approach for managing dentin hypersensitivity. Although various agents promote dentin remineralization, direct comparisons between theobromine, bioactive glass (BAG), and nano-hydroxyapatite (Nano-HAP) under simulated oral conditions remain limited. To fill this gap, this in vitro study aimed to evaluate and compare the effectiveness of these three treatments on exposed cervical dentin. The assessment focused on their chemical, morphological, and mechanical effects on dentin.

**Materials and methods:**

Forty-eight human dentin slabs were obtained from the cervical portions of twelve sound premolar teeth. Baseline Raman spectroscopy and VMH tests were done to exclude outliers. All specimens were treated with 6 % citric acid (pH 2.0) for 2 min to remove the smear layer. They randomly assigned to four groups (n = 12): artificial saliva (AS), theobromine, BAG, and Nano-HAP. Evaluations were conducted using Raman spectroscopy (phosphate peak intensity at 960cm^−1^), Vickers microhardness testing (VMH), and morphological assessment under scanning electron microscopy (SEM).

**Results:**

Theobromine, BAG, and nano-HAP groups demonstrated a statistically significant increase in Raman phosphate peak intensity (960cm^−1^) and Vickers microhardness values (p < 0.05), indicating surface remineralization. In contrast, the artificial saliva group exhibited a significant decrease in phosphate peak intensity and microhardness values (p < 0.05).

**Conclusion:**

All tested agents significantly enhanced the Raman phosphate peaks and microhardness values compared to the control. Nano-HAP showed the highest potential for promoting the remineralization of exposed dentin surfaces. Within the study's limitations, it can be concluded that theobromine, BAG, and nano-HAP are effective in occluding dentinal tubules.

## 1 Introduction

Dentin hypersensitivity (DH) is a short, sharp pain from exposed dentin in response to various stimuli, often in the buccal cervical region. Treatments include home-based and professional options, with home-use products being simpler and more cost-effective. Most treatments work by occluding dentin tubules or altering nerve response to reduce permeability.^1^

White crystalline powder theobromine (3, 7 dimethylxanthine) is an alkaloid that is easily found in cocoa and chocolate. Theodent Classic ®, a commercially available toothpaste containing theobromine instead of fluoride, was created to prevent and treat dental cavities. Bioactive glass (BAG) is biocompatible and promotes hydroxyapatite formation via calcium and phosphate release and cause sealing of the dentinal tubules and protects them from the damaging effects of the acidic environment by binding to collagen fibers. Nano-HAP, the main component of enamel and dentin, effectively occludes tubules due to its nano-scale size and block the flow of fluid.^2^, ^3^, ^4^

Raman spectroscopy and microhardness testing are reliable methods for evaluating mineral changes in dentin. We compared the effects of the three agents on demineralized cervical dentin using pH cycling. To the best of our knowledge, no prior study has compared them under these conditions. The null hypotheses stated that Raman peak intensity and microhardness would not significantly change across treatments.^5^^,^^6^

## 2 Material and methods

To evaluate the comparative effects of the tested agents on demineralized dentin, the following methodology was employed.

### 2.1 Sample collection and preparation

Using G Power 3.1.9.7 (Program written by Franz-Faul, Universitatit Kiel, Germany). Based on a medium to large effect size of 0.48 derived from a previous study,^7^ with a significance level (α) of 0.05, and a study power of 95 %, the total sample size required was calculated to be 48. This corresponded to 12 samples per group.^8^Using an ethics protocol approved by the health research committee (Ref NO. 849, November 23, 2023).

**Fig. 1:**
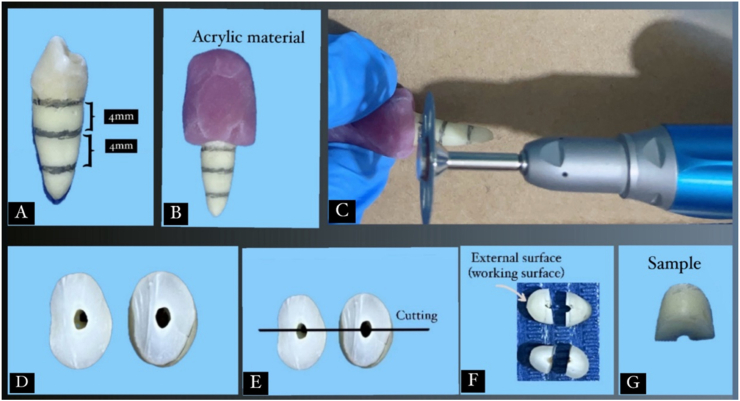
Sample sectioning procedure. A: The locations of three cut lines on the sound premolar marked with pencil, B: Investing the crown part with light cure acrylic material, C: Dentin discs sectioning, D: Dentin disc, E: Marking the location of sections on the dentin discs to be sectioned into two halves, F: Four dentin sample results from two disc, G: dentin sample.

**Fig. 2:**
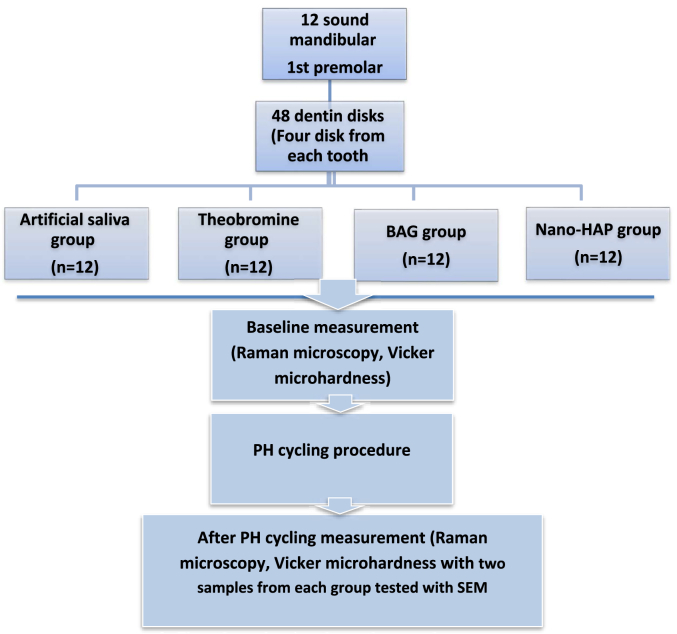
Flow chart showing the study procedures steps.

**Fig. 3:**
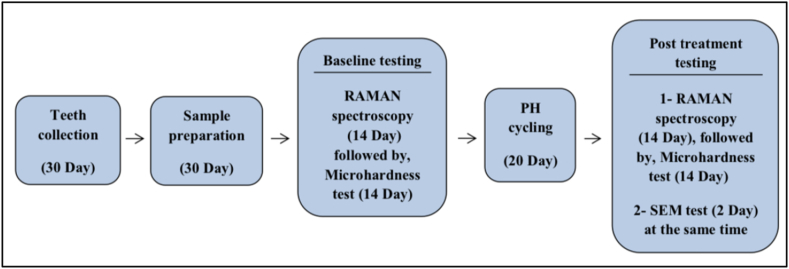
Timeline diagram of the experimental procedure.

Twelve sound lower first premolars extracted for orthodontic reasons were cleaned and polished with non-fluoridated pumice. Teeth with defects were excluded under 10× magnifications. Selected teeth were stored in 0.1 % thymol for 48 h, then in deionized water at 4 °C and used within one month. Only the cervical third of the roots was used. Two horizontal dentin discs were sectioned per tooth using a diamond disc under water cooling, yielding four specimens per tooth (4 3 × 2mm), for a total of 48 as demonstrated in the sample sectioning procedure (Fig. 1), baseline Raman spectroscopy and VMH tests were done to exclude outliers. All specimens were immersed in 6 % citric acid (pH 2.0) for 2 min to remove the smear layer that formed during specimen preparation leaving open dentin tubules.^9^^,^^10^ A detailed overview of the experimental procedure and its timeline is provided in Fig. 2, Fig. 3.

### 2.2 Experimental groups and randomization

The 48 specimens were randomly assigned to four groups (n = 12 each), with two from each group examined via SEM as showed in the flow chart:

1. Artificial saliva group: Demineralized dentin with no treatment.
2. Theobromine group: Treated with Theodent Classic toothpaste (Rennou, USA).
3. BAG group: Treated with Biomin Restore toothpaste (Dr Collins, USA).
4. Nano-HAP group: Treated with Davids Natural toothpaste (Premium natural toothpaste, USA).

### 2.3 PH cycling protocol

The samples were subjected to a pH cycling protocol to simulate demineralization and remineralization challenges. The detailed composition and timing schedule of the solutions are provided in the supplementary file.

### 2.4 Analytical methods

To reduce potential bias, group allocation was blinded from the examiner who conducted the microhardness testing, SEM analysis, and Raman spectroscopy throughout the study.

#### 2.4.1 Raman spectroscopy

40 dentin specimens underwent scans using a high-resolution confocal laser Raman Spectroscopy Parameters was performed using a Senterra spectrometer (Bruker Optics, Germany) operating in line scan mode. A 780nm near-infrared diode laser with a grating of 400 lines/mm was used. The laser power was set at 100mW, with an integration time of 30s per point. Spectra were acquired within the spectral range of 80–3800cm^−1^. Three scanning points were selected at the center of each dentin slab, with a 500μm distance between each point. Calibration was performed using a standard silicon wafer (520.7cm^−1^) prior to measurements. Post-acquisition, the spectra were processed using OPUS software (Bruker Optics, Germany). Quantitative analysis was performed using Origin (MicroCal Inc.) and Microsoft Excel 2019, by plotting spectral curves and measuring the intensity of the phosphate v1 peak at 960cm^−1^. Peak intensities were compared across all experimental groups.

#### 2.4.2 Microhardness testing

VMH has been measured for specimens by the same examiner with the use of the same calibrated machine. The Vickers hardness number was recorded automatically using the manufacturer's software, each sample was tested at three points, and mean values were calculated. Three indentations were done at middle of each surface with 500μm distance apart. The hardness profile was analyzed using a Vickers microhardness tester (TH715, Obsnap Instruments Sdn Bhd, Selangor, Malaysia). This tester utilized a diamond square-based pyramid indenter with a 100 gf (1N) load applied for 15s.^4^ Polishing of specimens ensured accurate measurements.

#### 2.4.3 SEM analysis

Two specimens representative of each group underwent drying and carbon-coating within a vacuum chamber (YKY SEM Coating System) set at a voltage of 30kV and intensity of 20mA. Each plate was coated for 4 min. Images were captured from the center of the surface of each dentin block at magnifications of 3000 and 8000 , with a working distance of 4.6mm (Fig. 2, Fig. 3).

### 2.5 Statistical analysis

Data description, presentation, and analysis were performed using SPSS software (version 26, IBM Corp., Armonk, NY, USA). Statistical analyses were categorized into descriptive and inferential approaches. The Shapiro–Wilk test was applied to assess the normality of data distribution. For inferential analysis, one-way analysis of variance ANOVA was conducted to evaluate statistically significant differences between group means. Post-hoc analyses were performed using Tukey's multiple comparison tests to identify significant differences between the groups, with a significance level of **(P** < 0.05).

## 3 Results

The outcomes of the experimental procedures are presented below, highlighting the chemical, mechanical, and morphological findings.

### 3.1 Chemical analysis (Raman spectroscopy)

The null hypothesis was rejected, as one-way ANOVA revealed a statistically significant difference in phosphate peak intensities among the tested groups (F = 127.454, p = 0.0001). Raman spectroscopic analysis following pH-cycling revealed significant differences (p = 0.000) in phosphate peak intensity among the groups, with the nano-HAP group maintaining the highest mean intensity (4409.10 ± 142.31), followed by the BAG (4054.25 ± 91.34), theobromine (4159.40 ± 116.60), and artificial saliva groups (3375.83 ± 128.37).

**Fig. 4:**
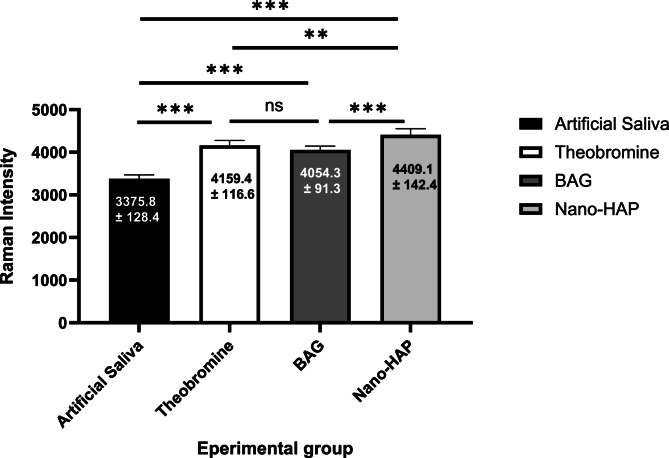
A graphical representation of Raman spectroscopy results showing the mean phosphate peaks intensity of dentin specimens after treatment with various remineralizing agents. An overall increase in remineralization was observed in all treated groups compared to the artificial saliva group, with nano-HAP showing the most pronounced effect.

**Fig. 5:**
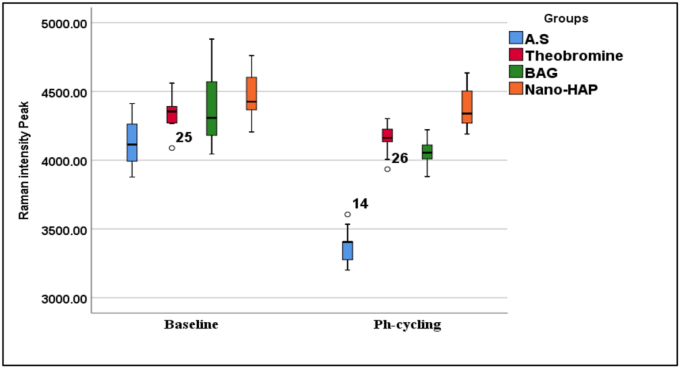
Box plots representing the distribution of Raman phosphate peaks intensity values for dentin specimens at baseline and after pH-cycling across different treatment groups.

**Fig. 6:**
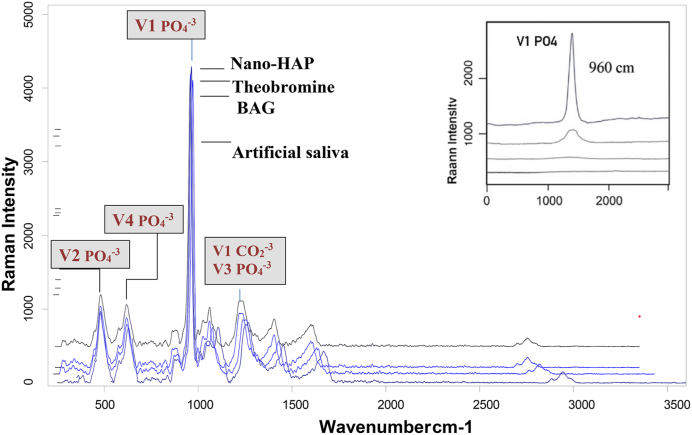
Representative Raman spectra of the artificial saliva, theobromine, BAG and nano-HAP groups after pH-cycling. The vibrational peak of v1- PO_4_^−3^at 960 cm-1 is the strongest signal of all Raman spectra and is highest in the Nano-HAP, followed by theobromine, BAG and artificial saliva groups respectively. V2- PO_4_^−3^at 450 cm-1, V3 - PO_4_^−3^at 1045 cm-1, V4 - PO_4_^−3^at 570 cm-1, V1- CO_2_^−3^at 1045 cm-1.

The percentage of change (%Δ) in intensity was greatest in the artificial saliva group (18.20 %), indicating substantial mineral loss, while the nano-HAP group showed the lowest change (1.22 %), and suggesting superior resistance to demineralization. Paired t-tests confirmed statistically significant differences within each group before and after pH-cycling (p = 0.000). Effect sizes (Cohen's d) were highest for the artificial saliva group (3.17), followed by 10.13039/501100002329BAG (2.61), theobromine (1.92), and nano-10.13039/501100015606HAP (1.06), which further supporting the protective effect of remineralizing agents, particularly nano-10.13039/501100015606HAP. An overview of the results can be seen in (Table 1, Fig. 4, Fig. 5, Fig. 6).

Post-hoc analysis using Tukey's HSD test revealed statistically significant differences between the artificial saliva group and all treated groups (p < 0.0001) as showed in the (Table 2), with the Nano-HAP group showing the highest mean difference (−1009.97), followed by theobromine (−783.57) and BAG (−678.42). Additionally, significant differences were also observed between BAG and Nano-HAP (p < 0.0001), and between theobromine and Nano-HAP (p = 0.001). However, the difference between theobromine and BAG was not statistically significant (p = 0.233). These findings suggest that Nano-HAP exhibited the most pronounced effect among the tested agents (Tables 1 and 2, Fig. 4, Fig. 5, Fig. 6).

**Table (1):** Intensities of Raman band (Mean ± SD) pre and post PH cycling with one ANOVA for comparison across the groups.

Phases		Groups				F	P value
		A.S^a^	Theobromine^b^	BAG^b^	Nano-HAP^c^		
Baseline	Minimum	3877.620	4088.500	4044.700	4205.300	2.478	0.076 NS
	Maximum	4411.580	4560.500	4880.600	4760.600		
	Mean	4133.070	4339.340	4372.350	4465.130		
	±SD	181.935	123.698	260.717	168.779		
	±SE	57.533	39.117	82.446	53.373		
Ph-Cycling	Minimum	3200.970	3934.500	3880.500	4190.50	127.454	**0.000 Sig.**
	Maximum	3605.120	4302.500	4221.000	4634.500		
	Mean	3375.832	4159.400	4054.250	4409.100		
	±SD	128.366	116.600	91.339	142.310		
	±SE	40.593	36.872	28.884	45.673		
% Δ		18.205	4.135	6.953	1.216		
Paired T test		10.023	8.267	3.339	6.075		
P value		**0.000**	**0.000**	**0.009**	**0.000**		
Cohen D		3.170	1.921	2.614	1.056		

NS: not significant, sig: significant.

**Table (2):** Tukey Honestly Significant Difference (Tukey HSD) for Raman peak intensities between each group.

Groups	Groups	Mean Difference	Std. Error	P value	95 % Confidence Interval	
					Lower Bound	Upper Bound
Artificial saliva	Theobromine	−783.56800-∗	54.44095	**0.000∗**	−930.1898-	−636.9462-
	BAG	−678.41800-∗	54.44095	**0.000∗**	−825.0398-	−531.7962-
	Nano-HAP	−1009.96800-∗	54.44095	**0.000∗**	−1156.5898-	−863.3462-
Theobromine	BAG	105.15000	54.44095	0.233^	−41.4718-	251.7718
	Nano-HAP	−226.40000-∗	54.44095	**0.001∗**	−373.0218-	−79.7782-
BAG	Nano-HAP	−331.55000-∗	54.44095	**0.000∗**	−478.1718-	−184.9282-

∗ = significant at p < 0.05, ^ = not significant at p > 0.05, CI: Confidence Interval.

### 3.2 Surface microhardness

The null hypothesis was rejected, as one-way ANOVA revealed no statistically significant difference in the baseline microhardness values among the tested groups (F = 1.448, p = 0.245), indicating similar initial surface hardness across all specimens. However, following pH-cycling, a statistically significant difference was observed (F = 23.019, p = 0.000), confirming that the type of treatment had a substantial impact on dentin microhardness under acidic conditions. Post-cycling measurements showed that the artificial saliva group exhibited the lowest mean microhardness value (38.915 ± 2.092), indicating pronounced demineralization. In contrast, the Nano-HAP group showed the highest retention of hardness (50.280 ± 5.250), followed by Theobromine (46.690 ± 2.703) and BAG (41.550 ± 4.388).

The percentage reduction in microhardness (%Δ) further emphasized the protective potential of the tested agents. The Artificial Saliva group showed the greatest decrease (26.985 %), whereas BAG (5.680 %) and Nano-HAP (5.580 %) showed minimal reductions, indicating strong resistance to acidic challenge. The Theobromine group showed an intermediate reduction (11.989 %). Cohen's D values indicated large effect sizes, especially in the Artificial Saliva group (3.153), followed by BAG (2.859), Theobromine (1.970), and Nano-HAP (1.549). These findings confirm the effectiveness of remineralizing agents in preserving dentin microhardness following acidic challenge, with Nano-HAP and BAG exhibiting the highest efficacy. Theobromine also showed significantly higher values than the artificial saliva group (p < 0.001), while its difference with BAG was not significant (p = 0.211). A summary of the findings is illustrated in (Table 3).

Post-hoc comparisons using Tukey's HSD test were conducted to determine pairwise differences in Vickers hardness values between the groups after pH cycling as showed in (Table 4). The Artificial Saliva group showed statistically significant lower hardness values compared to both the Theobromine group (mean difference = −7.775, p = 0.000) and the Nano-HAP group (mean difference = −11.365, p = 0.000), while the difference with the BAG group was not statistically significant (mean difference = −2.634, p = 0.313). The Theobromine group exhibited a significantly higher microhardness compared to the BAG group (mean difference = 5.141, p = 0.008). However, the difference between Theobromine and Nano-HAP was not statistically significant (mean difference = −3.590, p = 0.098).

**Figure (7):**
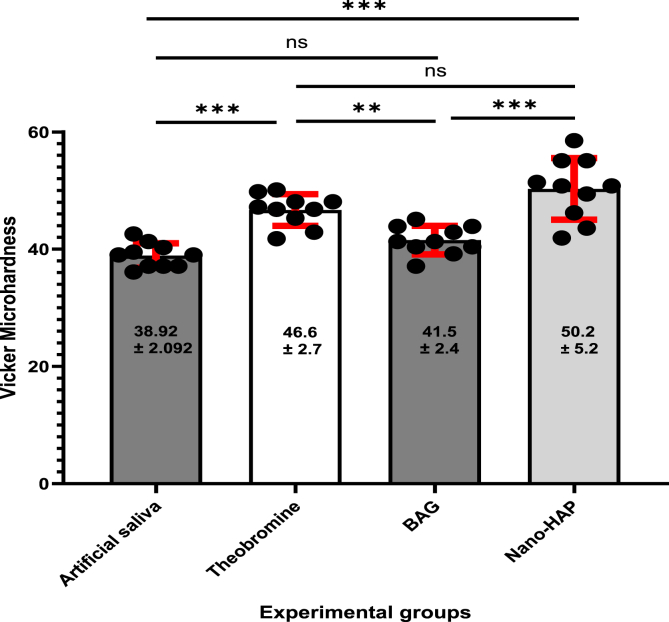
A graphical representation of Vickers microhardness results showing mean the means surface hardness values of dentin specimens following treatment with various remineralizing agents. All treated groups demonstrated increased remineralization compared to the artificial saliva group, with nano-HAP exhibiting the most significant enhancement.

**Figure (8):**
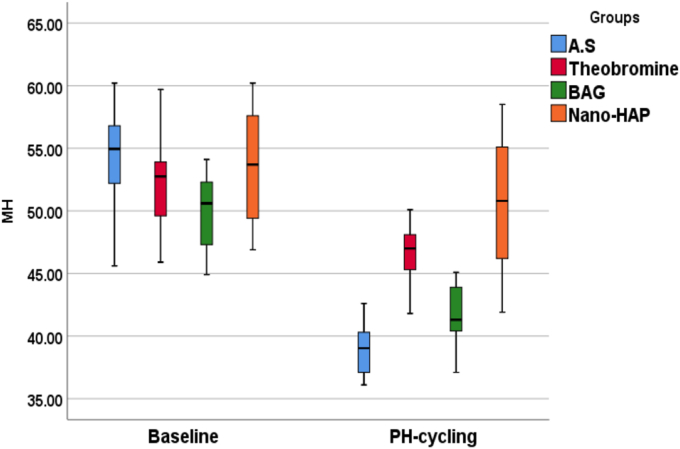
Box plots representing the distribution of Vickers microhardness (VHN) values for dentin specimens at baseline and after pH-cycling between the groups.

A highly significant difference was observed between BAG and Nano-HAP groups (mean difference = −8.731, p = 0.000), with the Nano-HAP group showing superior hardness values (Table (3), Table (4), Figure (7), Figure (8)).

**Table (3):** Minimum, maximum, mean and standard deviation of Vickers microhardness test peaks in all groups.

		Groups				F	P value
		A.S^a^	Theobromine^b^	BAG^a^	Nano-HAP^b^		
Baseline	Minimum	45.600	45.900	44.900	46.900	1.448	0.245 NS
	Maximum	60.200	59.700	54.100	60.200		
	Mean	53.700	52.600	50.070	53.260		
	±SD	4.712	4.307	3.043	4.766		
	±SE	1.490	1.362	0.962	1.507		
Ph-Cycling	Minimum	36.100	41.800	37.100	41.900	23.019	**0.000 Sig.**
	Maximum	42.600	50.100	45.090	58.500		
	Mean	38.915	46.690	41.549	50.280		
	±SD	2.092	2.703	2.438	5.250		
	±SE	0.662	0.855	0.771	1.660		
% Δ		26.985	10.910	16.879	5.680		
Paired T test		9.042	4.897	9.971	6.229		
P value		**0.000**	**0.001**	**0.000**	**0.000**		
Cohen D		3.153	1.970	2.859	1.549

**Table (4):** Tukey Honestly Significant Difference (Tukey HSD) for VHN (kg/mm2) between each group.

Group	Groups	Mean Difference	Std. Error	Sig.	95 % Confidence Interval	
					Lower Bound	Upper Bound
Artificial saliva	Theobromine	−7.77500-∗	1.50325	**0.000∗**	−11.8236-	−3.7264-
	BAG	−2.63400-	1.50325	0.313^	−6.6826-	1.4146
	Nano-HAP	−11.36500-∗	1.50325	**0.000∗**	−15.4136-	−7.3164-
Theobromine	BAG	5.14100∗	1.50325	**0.008∗**	1.0924	9.1896
	Nano-HAP	−3.59000-	1.50325	0.098^	−7.6386-	0.4586
BAG	Nano-HAP	−8.73100-∗	1.50325	**0.000∗**	−12.7796-	−4.6824-

∗ = significant at p < 0.05, ^ = not significant at p > 0.05.

### 3.3 Correlation between phosphate and VHN

**Figure (9):**
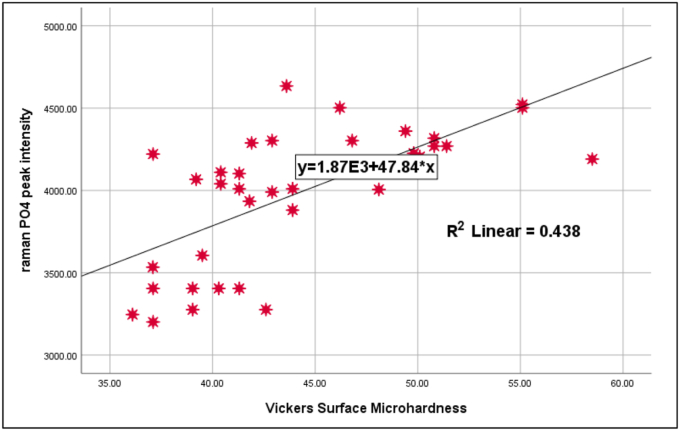
A scatter plot and a regression line (R) demonstrating the presence of a logarithmic regression relationship (p < 0.05) between the phosphate peak ratios and corresponding Vickers microhardness number (VHN) in each group.

Pearson correlation analysis across all groups revealed a statistically significant positive correlation between phosphate Raman peak intensity and Vickers microhardness values (r = 0.600, p = 0.000), suggesting that increased mineral content is generally associated with higher surface hardness. However, when analyzed within each group individually, no statistically significant correlations were observed (p > 0.05) as demonstrated in (Table 5). The peak intensity of phosphate ν1 of the selected points in each dentin sample were calculated and plotted against Vickers hardness numbers (VHN) as a scatter diagram, as shown in (Fig. 9).

**Table 5:** Person correlation PO_4_^−3^ 960 cm-1: VHN for all groups after pH-cycling (p < 0.05).

Groups	Person test	
	r	P
A.S	0.086	0.813
Theobromine	0.247	0.492
BAG	−0.610	0.061
Nano-HAP	−0.280	0.434
Total	0.661	**0.000**

### 3.4 Morphological observations

**Figure (10):**
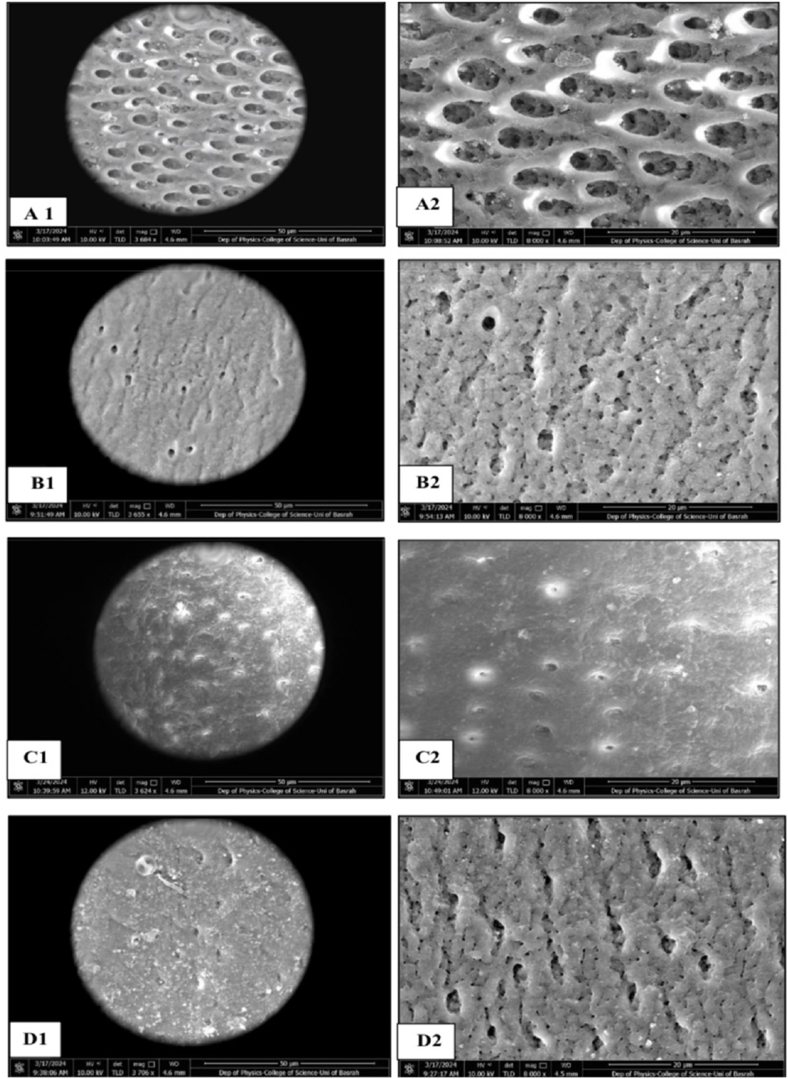
SEM image for all groups of the study. Images A1-A2: Artificial saliva dentin samples: showing the typical morphology of the dentin surface after removal of the smear layer, B1-B2: Theobromine samples: Demonstrated deposition of precipitate layer with heterogeneous distribution on dentin surface and precipitative occlusion of dentin tubules, C1-C2: BAG group samples: Dentin surface characterized by irregular dense packed crystal-like layer deposits leaving predominantly higher number of tubular occlusion, and D1-D2: Nano_HAP sample: showing a predominantly higher number of tubular occlusion and partial coverage of the dentinal surface with film or precipitate. (1: 3000X, 2: 8000X).

SEM images revealed notable differences in dentinal surface morphology among the tested groups following pH-cycling (Fig. 10). The Artificial Saliva group exhibited a heavily demineralized surface with wide-open dentinal tubules, indicating minimal protective effect. In contrast, the Theobromine group showed partial tubule occlusion and a relatively smoother surface. Samples treated with BAG demonstrated more substantial occlusion of dentinal tubules, with the presence of a granular surface layer suggestive of mineral deposition. The Nano-HAP group exhibited the most uniform and dense occlusion, with nearly complete coverage of the tubules by a continuous mineral-like layer. These morphological observations support the quantitative findings.

## 4 Discussion

The findings are interpreted and discussed in the context of previous literature, with emphasis on the clinical relevance and implications of each tested agent.

Dental hypersensitivity occurs when dentinal tubules are exposed to the oral environment due to root surface exposure from gingival recession, periodontal disease, or treatment, as well as enamel loss through abrasion or erosion.^11^ Since gingival recession is the leading cause of dentin hypersensitivity, exposing cervical dentin tubules to the oral cavity, we considered cervical dentin more clinically relevant than coronal dentin in this study.^10^ We conducted this study in controlled in vitro conditions, which may not fully simulate the complex oral environment.

### 4.1 Remineralization effects

Raman microspectroscopy, known for its 97 % sensitivity and 100 % specificity, was used to analyze the molecular composition of mineralized dental tissues. A key peak observed in all spectra was the phosphate ν1 band at 960cm^−1^, which indicates the mineral status of dentin 12. Increased peak intensity suggests successful remineralization due to changes in crystallite morphology and higher calcium and phosphate concentrations. Among all groups, the nano-hydroxyapatite (nano-HAP) group exhibited the highest Raman intensity values, while the artificial saliva (AS) group showed the lowest.^12^^,^^13^ Therefore, the first null hypothesis was rejected.

The strong Raman signal at 960cm^−1^ was also associated with amorphous calcium phosphate, which requires less energy to absorb due to its disordered structure. Peak intensity correlates directly with crystallinity; hence, the high intensity in the nano-HAP group reflects enhanced crystallization.^14^

In Theodent classic toothpaste group, which containing theobromine material that correspondent to theobromine solution at a concentration of 500mg/L, in which the theobromine (C7H8N4O2) molecule binds to crystals HA then replace the crystalline ions. The high-electronegativity of nitrogen and oxygen molecules tends to attract ions (Ca) and phosphates (PO4) with low electronegativity level. Besides that, H + methyl group dissolve easier and replaced by calcium-phosphate.^15^ Those two thing are possible for theobromine molecule to attract calcium and phosphate ions, so that the deposition of calcium and phosphate occurs to form a new hydroxyapatite crystal called Theobromine apatite [Ca10(PO4)6 (OHC7H8N4O2)].^16^ The contribution of calcium, phosphate, and hydroxyl ions present in saliva to apatite deposition is fundamental. This coincides with the Sadeghpour et al., 2007 revealed that theobromine makes calcium and phosphate to merge into a crystal unit that is a fourfold bigger than hydroxyapatite.^17^ Thorn et al. (2020) and Lippert (2017) reported findings that contradict our results, showing no significant remineralization effect of theobromine. This discrepancy may arise from methodological differences, particularly the use of continuous exposure models versus pH cycling in the present study and using lower concentration of theobromine respectively.^18^^,^^19^

The unique chemical composition and structure of bioactive glasses enhance the Raman signals in the samples that capable of rapid dissolution and breakdown of silica network, accompanied by the release of Ca2+, PO4 3- and Si4+ occurs at the glass surface within a demineralized dentin matrix which promote mineral deposition.^20^, ^21^, ^22^ The release of ions causes a pH rise and induces apatite formation in dental hard tissues. Besides, the sodium (Na+) and hydrogen ions (H+) or hydronium ion (H3O) exchanges, and rapid release of Ca+ ^2^and PO4-^3^ from BAG may contribute, together with the Si(OH)4 condensation and the apatite precipitation, this could indicate that the presence of BAG particles within the demineralized dentin matrix can actively initiate and develop true functional crystallization and remineralization within samples of this group.^23^ The increased intensity of the Raman peaks corresponding to more hydroxyapatite formation can be attributed to enhanced remineralization. This occurs because the solution is saturated with calcium and phosphate, which facilitates the deposition of minerals back into the tooth.^24^ Finally, A polycondensed silica-rich gel layer forms on the glass bulk, which may act as a template for apatite nucleation, growing by incorporating more Ca2+ and PO4 3- from the surrounding fluid. Therefore, the formation of apatite on the glass surface is influenced by the concentration of effective ions like Ca2+, PO4 3-, and OH- released into the reaction medium, which in turn depends on their concentration in the bioactive glass and its reactivity. The increase in the Raman phosphate intensity v1 at 962 cm-1, corresponding to hydroxyapatite, following bioactive glass treatment, is due to the reaction mechanism involving dissolution, leaching, and precipitation.^25^

When the pH rises, the presence of Si-O creates a template that promotes mineral precipitation. Due to these deposits, dentin may become more resistant to acid dissolution during the repair process, even when challenged by acid.^26^ Increase in the concentrations of calcium and phosphate at the dentin surface of 10.13039/501100002329BAG group that support these expectation even after pH-cycling, silicon ions were still observed to be adhered to the surface. A clinical in situ investigation by Sun et al. (2014) that verified following four weeks of toothpaste application, The new minerals deposited on a bovine insert, which included calcium silicate and sodium phosphate salts, strongly supports this conclusion.^27^ Another study that concludes bioactive glass-containing toothpaste significantly reduced dentine permeability after the 7-day treatment and showed, under SEM, excellent resistance to acid challenge compared to the other groups. ATR/FTIR and EDX revealed increased mineral content after treatment with Novamin^28^.

Hydroxyapatite is a naturally occurring mineral that makes up a significant portion of tooth structure. Nano-HAP, due to its small particle size and high surface area-to-volume ratio, penetrates dentin tubules and microscopic defects more effectively. Nano-hydroxyapatite forms a protective layer on the external surface of dentin in root specimens, resulting in the occlusion of dentin tubules by mineral hydroxyapatite, thus reducing dentinal permeability, and preventing fluid disturbance within the tubules and decreasing dentinal hypersensitivity.^29^^,^^30^ Nano-HAP particles attract an enormous amount of calcium and phosphate ions from the surrounding solutions (saliva) to the tooth structure, thus promoting crystal integrity and growth.^31^ When nano-hydroxyapatite material is subjected to Raman microscopy, several effects can be observed such as enhanced Raman scattering due to the small size of nano-hydroxyapatite particles that improve the spatial resolution of Raman microscopy and allows for the characterization of nanoscale features which lead to stronger Raman signals, making it easier to detect and analyze, this means that even small concentrations or variations in the sample can be detected more easily confirming its remineralization capability.^32^ David's natural toothpaste was used in the study, nano-Hydroxyapatite (calcium phosphate) which is the key active ingredient of the toothpaste, providing remineralization benefits to potentially reduce sensitivity.^33^

Several studies have highlighted the promising role of nano-hydroxyapatite in dentin remineralization. Tschoppe et al. (2011) conducted an in vitro study to evaluate the remineralization effects of nano-HAP containing toothpaste on demineralized bovine enamel and dentin, comparing it with conventional amine fluoride toothpaste. The results demonstrated that toothpastes containing nano-HAP significantly enhanced remineralization, particularly in dentin, with higher concentrations yielding more pronounced effects.^34^ Double-blind, randomized clinical trial done by Amaechi et al. (2021) compared toothpastes containing 10 % and 15 % n-HAp, with and without potassium nitrate, to a commercial desensitizing dentifrice containing calcium sodium phosphosilicate (CSPS). Over eight weeks, all test toothpastes significantly reduced dentin hypersensitivity, with 15 % n-HAp and 10 % n-HAp with potassium nitrate showing consistent effectiveness, comparable to the CSPS-containing toothpaste.^35^ In contrast to study reporting strong remineralizing effects of nano-hydroxyapatite, Najibfard et al. conducted an in vitro study comparing toothpaste formulations containing 5 % and 10 % n-HAP. The findings showed no significant difference in the remineralization potential between the two concentrations. This suggests that simply increasing the concentration of nano-HAP does not necessarily enhance its effectiveness in promoting dentin remineralization, indicating a possible saturation point beyond which no additional benefit is achieved.^36^

The Raman-scattering spectra of all samples exhibited a sharp, dominated and strong band at 960 cm−1 which was associated with the amorphous calcium phosphate suggesting that less energy is required for the disordered calcium to absorb energy. The intensity depends directly on the sample crystallinity, so the highest degree of crystallinity of the nano-HAP samples appears to be achieved after calcination.^14^^,^^32^ The large effect sizes of Raman peek intensity observed in this study highlight the clinical relevance of the remineralizing agents tested. Nano-HAP and theobromine, in particular, demonstrated very large effects compared to artificial saliva.

### 4.2 Mechanical properties

Microhardness is directly related to the amount of hydroxyapatite and serves as an indirect marker of mineral gain or loss. Vickers microhardness testing was employed due to its suitability for curved and demineralized surfaces.^37^^,^^38^

In the measurement of surface microhardness, many studies have suggested various methods, the most common of which are the Vickers microhardness and Knoop microhardness tests.^39^ In this study, the Vickers microhardness test was used. Studies have shown that the Vickers hardness device is more useful in calculating the surface microhardness of teeth samples because the indenter of this device can be used on demineralized specimens and is less affected by teeth curvature. All teeth samples used for microhardness evaluation were polished because any tilting or non-flat surface might produce a very long indentation and hence a lesser hardness value. Consequently, flat and smooth teeth surface was essential in this test.^38^ The results showed that the micro-hardness of demineralized dentin surfaces significantly decreased compared to the experimental groups, indicating a loss of minerals and this is in accordance with study done on the root dentin, which conclude that the citric acid reduce the microhardness from 52.50 VHN to 47.30 VHN due to the loss of peritubular and intertubular dentin. This could be attributed to the fact that all the samples have been subjected to the same demineralizing solution and immersion time for the aim of standardization.^40^^,^^41^

When treated the demineralized dentin with theobromine material, microhardness values increased but remained lower than those of sound dentin. In a prior study conducted by Farooq et al., it was proposed that theobromine could have permeated the micro-tunnels of hydroxyapatite (HA), inducing internal stress that resulted in increased resistance to indentation consequently, higher microhardness,^42^ this agree with the result of a study done on enamel which conclude that following exposure to 200mg/L theobromine gel, there was an increase in hardness observed in the demineralized enamel surfaces.^43^ The alteration of dentin hardness is higher in theobromine group than BAG group, This is due to theobromine apatite crystals which is formed by theobromine molecules, have a crystal size 4 times larger than hydroxyapatite crystals^44^

In the current study, Biomin Restore toothpaste, containing chloro calcium phosphosilicate was utilized. This composition potentially led to the deposition of these minerals onto the dentin surface, ultimately resulting in the hardening of the dentin surface. The researchers attributed this hardening to the chemical composition of BAG and the subsequent formation of mineral deposits within the dentinal tubules.^45^

The findings of the current study were supported by study conducted by Jones et al. (2015); they discovered that toothpaste containing NovaMin resulted in increased dentin microhardness after 5 and 10 days of brushing twice daily. They attributed this dentin hardening to NovaMin's chemical composition, combined with the presence of fluoride in the toothpaste formulation.^46^ In contrast, a contradictory study conducted by Sauro et al. (2011) suggested that there was no change in dentin surface microhardness either immediately after applying toothpaste containing BAG onto demineralized dentin specimens or following storage in a remineralizing solution. The authors proposed that a single application of BAG-containing paste is inadequate to trigger dentin remineralization.^47^

A recent in vitro study by Mehrjoo et al. (2024) investigated the impact of nano-hydroxyapatite containing toothpaste on the microhardness of enamel surfaces subjected to erosive challenges. The results revealed that enamel surfaces treated with nano-HAP toothpaste exhibited stable microhardness levels, than those treated with fluoride gel or artificial saliva. These findings highlight the protective and remineralizing effect of nano-HAP in preserving enamel surface hardness under acidic conditions. Prior studies also support efficacy of nano-10.13039/501100015606HAP over 10.13039/501100002329BAG in remineralizing early carious lesions.^30^^,^^48^, ^49^, ^50^

The large effect sizes of surface microhardness observed in this study highlight the clinical relevance of the remineralizing agents tested. Nano-HAP and theobromine, in particular, demonstrated very large effects compared to artificial saliva, indicating their substantial capacity to enhance dentin microhardness.

### 4.3 Clinical implications

Although we conducted this study under controlled laboratory conditions, its findings align with a growing body of in vivo and in situ evidence that enhances its clinical relevance. Researchers have investigated that theobromine as a promising agent capable of occluding dentinal tubules and reducing hypersensitivity. In one in situ study, participants wore palatal appliances that held dentin blocks, allowing natural exposure to oral conditions such as saliva, temperature changes, and microbial activity.^10^ This setup produced results with strong clinical relevance and supported theobromine's potential effectiveness in the management of dentin hypersensitivity.

A randomized clinical trial at Al-Azhar University (2020) evaluated the effectiveness of theobromine, Remin Pro®, and their combination. Theobromine alone significantly reduced sensitivity, performing similarly to Remin Pro®. The combined formulation achieved the greatest reduction, indicating a possible synergistic effect. These results support that theobromine as a viable desensitizing agent, although additional long-term trials are necessary.^51^

Several clinical trials also confirm the benefits of bioactive glass (BAG)-based formulations. Zhang et al. (2024) conducted a 6-week randomized, double-blind controlled trial and found that toothpaste with HX-BGC bioactive glass-ceramic significantly reduced DH at 2, 4, and 6 weeks, outperforming placebo and NovaMin^52^. Rajendran et al. (2019) showed that BioMin F (fluorocalcium phosphosilicate) produced greater reduction in DH than arginine-calcium carbonate over four weeks.^53^ Orsini et al. (2010) conducted a double-blind randomized trial and confirmed that NovaMin-based toothpaste reduced sensitivity rapidly and maintained the effect over time.^54^

Nano-hydroxyapatite has also shown strong clinical performance. In a 3-month randomized trial, nano-HAP toothpaste sustained its desensitizing effect, unlike other dentifrices whose impact diminished after one month.^55^ In 2017, researchers compared nano-HAP with 8 % arginine-based toothpaste and found that both significantly reduced sensitivity within 5 min, 1 week, and 4 weeks.^56^ Therefore, the potential of a dentifrice to occlude dentin tubules in real-life clinical use may not be fully generalized from laboratory findings, especially when the dentinal tubules were not filled with natural dentinal fluids, as reported in previous studies. Further in vivo investigations are needed to confirm their clinical applicability.^10^

### 4.4 Study limitations

While the study yielded insightful results and demonstrated significant changes in dentin structure and mineral content using Raman microspectroscopy and Vickers microhardness testing, several limitations must be acknowledged to guide future research.

1. Although the study uses a clinically relevant pH-cycling model, the experiments were conducted in a controlled laboratory environment. Thus, the results may not fully replicate the complex conditions of the oral cavity, such as variations in saliva composition, patient compliance, and oral microbiota.
2. The effectiveness of the tested agents (Theobromine, BAG, and Nano-HAP) has not yet been confirmed in clinical settings. Further in vivo or clinical studies are needed to validate these findings on actual patients.
3. Despite rigorous sampling and standardization, individual variations in dentin composition (e.g., age, tooth type, previous fluoride exposure) could still influence results and limit generalizability.
4. While the 20-day model is a strength in terms of duration for an in vitro design, longer-term effects of the agents on dentin structure and potential cytotoxicity cannot be assessed without extended follow-up or biological testing.
5. The study primarily evaluates surface changes; subsurface remineralization or deeper structural integration of the agents is not fully assessed.
6. Lack of intra- and inter-observer reliability evaluation for the measurements. Although all assessments were performed by trained and calibrated examiners, future studies should incorporate reliability analysis to ensure consistency and reproducibility.

## 5 Conclusions

Based on the findings, the following conclusions can be drawn regarding the comparative efficacy of the tested desensitizing agents:

All tested materials improved both Raman peak intensities and microhardness under pH cycling conditions. Nano-hydroxyapatite showed the highest remineralization potential, followed by theobromine and bioactive glass. Within the study's limitations, all agents demonstrated potential to occlude dentinal tubules and enhance dentin mineralization.

## Patient conset

This in vitro study, so there is no patient conset.

## Ethical approval

Using an ethics protocol approved by the health research committee (Ref NO. 849, November 23, 2023).

## Funding

This is a self-funded study without any external funding done for the same.

## Declaration of Competing interest

There are no conflicts of interest to declare.
