# Supplementary material for: Morphological & chemo mechanical analysis of exposed cervical dentin treated with three different desensitizing pastes (comparative in vitro study)
Source: J Oral Biol Craniofac Res. 2025 Jul 7;15(5):982–91. doi: 10.1016/j.jobcr.2025.06.018 (PMC12273211; doi:10.1016/j.jobcr.2025.06.018)
Supplement: Multimedia component 1 [file mmc1.docx]

**2. Material and methods**

**2.2 Experimental Groups and Randomization**

The 48 specimens were randomly assigned to four groups (n=12 each), with two from each group examined via SEM.

1. Artificial saliva group: Demineralized dentin with no treatment.

2. Theobromine group: Treated with Theodent Classic toothpaste (Rennou, USA) (EXP Date 11-2025) (Fig. 2-A, Table 3), typical concentration of theobromine in toothpaste around 0.2% by weight.

3. BAG group: Treated with Biomin Restore toothpaste (Dr Collins, USA) (EXP Date 05-2025) (Fig. 2-B, Table 3) typical concentration of BAG in toothpaste around 5% to 15% by weight.

4. Nano-HAP group: Treated with Davids Natural toothpaste (Premium natural toothpaste, USA) (EXP Date 09-2025) (Figure 2-C, Table 3) typical concentration of nano-HAP in toothpaste around 10% by weight.

**
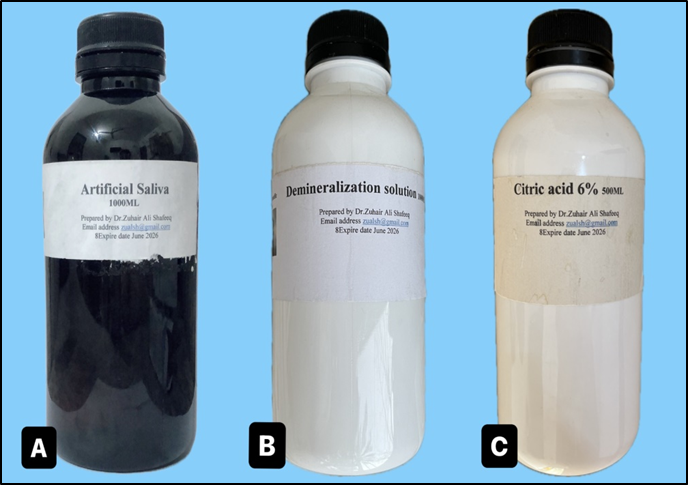
**

**Figure 1: (A: Artificial saliva, B: Demineralization solution, C: Citric acid 6%).**


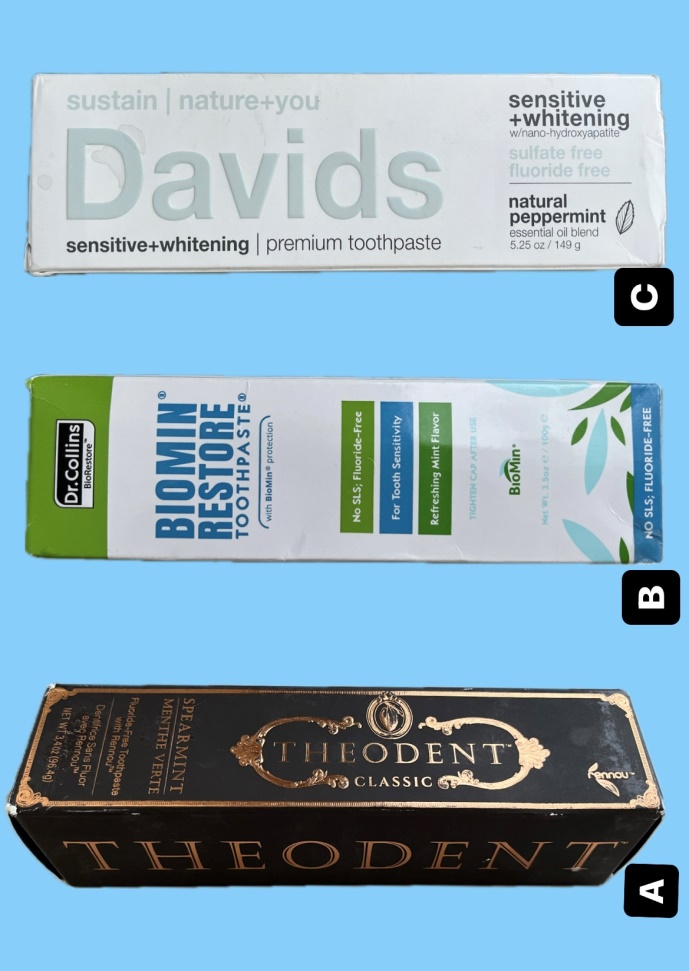


**Figure 2: (A: Theodent toothpaste, B: Biomin restore ttothpaste, C: Davids toothpaste).**

**2.3 pH Cycling Protocol**

Artificial saliva will be used to create slurry of each paste, as it acts as a remineralizing solution. A mixture of one part paste (9 g) to three parts remineralizing solution (27 mL) will be thoroughly blended in a beaker using a magnetic stirrer for 4 minutes. The samples have been placed in pH-cycling system solutions for 20 days. The daily cycle treatment plan include: four 2-min dentifrice slurry treatment periods and a 4-h acid challenge in the demineralization solution. Specimens were kept in artificial saliva (The remineralizing solution) the rest of the time. The demineralization treatment was not stirred, unlike the dentifrice slurry and saliva treatments. The specimens were gently washed under running deionized water following each treatment. After that, all specimens were reapplied in artificial saliva. Fresh remineralizing solution will be replaced three times per week, while Fresh demineralization solution was changed twice per week. All experimental procedures, including sample preparation, treatment application, and analytical measurements, were conducted under controlled laboratory conditions. The ambient room temperature was maintained at 23 ± 2°C, with a relative humidity of approximately 50 ± 5% with stirring movement 250 rpm then, assessment using Raman micro-spectroscopy, Vickers microhardness testing and scanning electron microscope (1) .


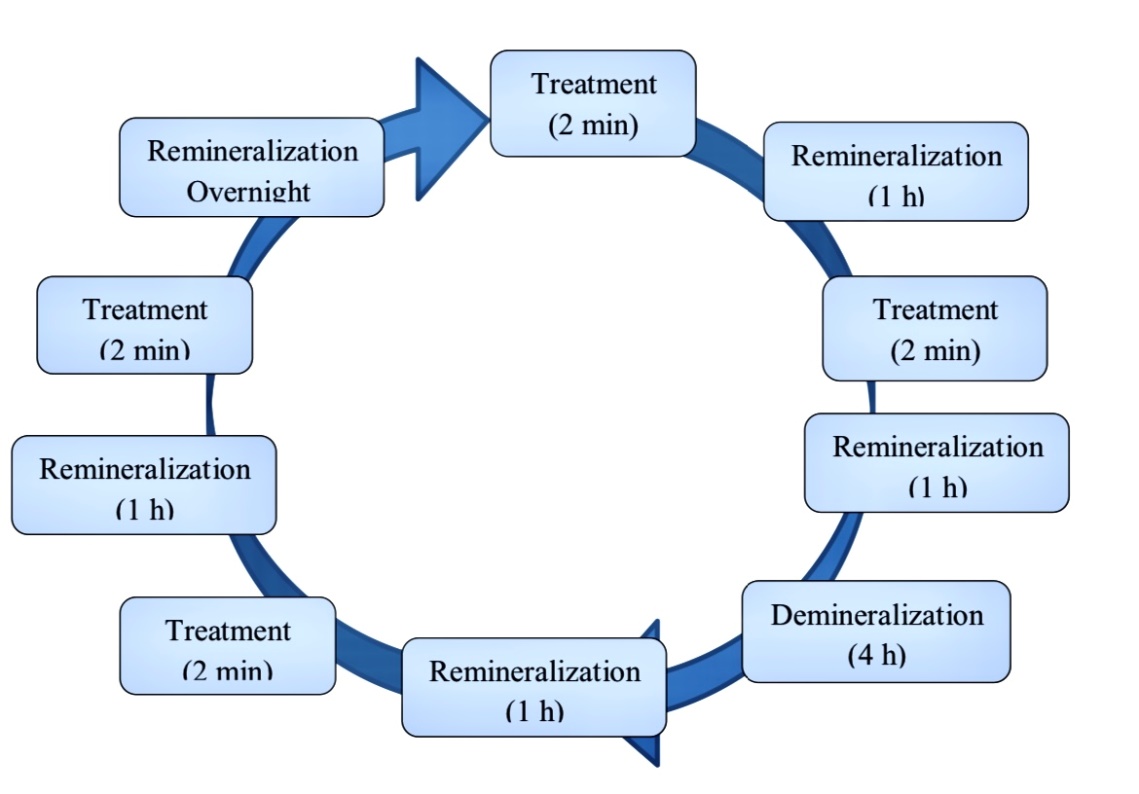


Figure 1: schematic diagram of pH cycling protocol


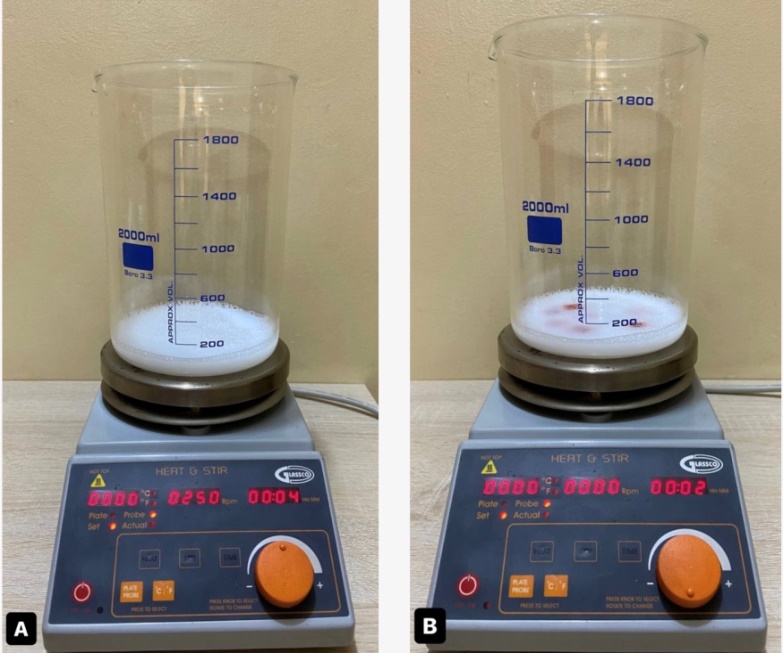


Figure 2: (A: Paste slutty mixed by magnetic stirrer, B: Dentin sample in the paste slurry).

Table (1): Treatment schedule for the pH cycling phase (1).

| The treatment schedule does not commence on the first day; instead, the specimens are stored in saliva under constant gentle rotation to facilitate the formation of an artificial pellicle-like layer | |
| --- | --- |
| **Duration** | **Phase of pH cycling** |
| 2 min (8:00 – 8:02 a.m.) | Treatment |
| 1 h (8:02 – 9:00 a.m.) | Artificial saliva (remineralization) |
| 2 min (9:00 – 9:02 a.m.) | Treatment |
| 1 h (9:02 – 10:00 a.m.) | Artificial saliva |
| 4 h (10:00 a.m. – 2:00 p.m.) | Demineralization solution |
| 1 h (2:00 – 3:00 p.m.) | Artificial saliva |
| 2 min (3:00 – 3:02 p.m.) | Treatment |
| 1 h (3:02 – 4:00 p.m.) | Artificial saliva |
| 2 min (4:00 – 4:02 p.m.) | Treatment |
| Overnight (4:02 p.m. – 8:00 a.m.) | Artificial saliva |

Table (2): Demineralization/remineralization solutions.

| Demineralization solution (pH 4.5) | Remineralization Solution (pH 7.0)  (Artificial saliva) |
| --- | --- |
| Calcium: 2.0 mmol/L 0.4723 g/L  Ca(NO3)2 - 4H2O, Mwt = 236.16.  Phosphate: 2.0 mmol/L, 0.27 g/L  KH2PO4 (Mwt = 136.09)  Acetic acid: 75.0 mmol/L, 4.5083 g/L  CH2COOH: Mwt = 60.05 | Calcium: 1.5 mmol/L 0.3542 g/L  Ca(NO3)2 - 4H2O, Mwt = 236.16  Phosphate: 0.9 mmol/L, 0.1225 g/L  KH2PO4: Mwt = 136.09  KCL Mwt = 74.55  NaCacodylate: 20 mmol/L, 4.28 g/L  NaC2H6AsO2-3H2O, Mwt = 214 |

Table (3): Materials used as remineralizing agents

| Material | Composition |
| --- | --- |
| Theodent classic toothpaste | Rennou (Theobromine, Calcium Acetate, Disodium Phosphate), Aqua/Water/Eau, Hydrated Silica, Xylitol, Sorbitol, Glycerin, Sodium Lauroyl Sarcosinate, Xanthan Gum, Titanium Dioxide (CI 77891), Citric Acid, Sodium Bicarbonate, Sodium Benzoate, Sodium Chloride, Stevioside, Mentha Viridis (Spearmint) Leaf Oil, Vanilla Planifolia Flower Extract Limonene |
| BioMin restore toothpaste | Chloro Calcium Phospho Silicate, Glycerin, PEG (Polyethylene Glycol), Silica, Cocamidopropyl Betaine, Sodium Methyl Cocoyl Taurate, Titanium Dioxide, Carbopol (Carbomer 974P), Flavor, Potassium Acesulfame |
| Davids natural toothpaste | Calcium Carbonate (Limestone Abrasive), Vegetable Glycerin, Purified Water, Sodium Bicarbonate (Baking Soda), Xylitol (Birch Derived), Hydrated Silica, Sodium Cocoyl Glutamate, Carrageenan (Seaweed Derived), Mentha Piperita (Peppermint) Oil, Mentha Viridis (Spearmint) Leaf Oil, Pimpinella Anisum (Anise) Seed Extract, Gaultheria Procumbens (Wintergreen) Leaf Oil, Stevia (Leaf Extract). |

**3. Results**

**3.1. Normality of Raman data**

The results of the normality, by using Shapiro-Wilk test, revealed that the intensities of Raman phosphate peaks (v1) are normally distributed as there was no significant difference among all groups (p˃0.05); as shown in (Table 4)

**Table 4: The Normality of phosphate peaks using Shapiro-Wilk Test.**

| Groups | Baseline | | | Ph-cycling | | |
| --- | --- | --- | --- | --- | --- | --- |
|  | Statistic | df | P value | Statistic | df | P value |
| A.S | 0.962 | 10 | 0.810 | 0.923 | 10 | 0.385 |
| Theobromine | 0.955 | 10 | 0.728 | 0.913 | 10 | 0.300 |
| BAG | 0.946 | 10 | 0.626 | 0.964 | 10 | 0.826 |
| Nano-HAP | 0.966 | 10 | 0.856 | 0.921 | 10 | 0.368 |

**3.2 Normality of Vickers Microhardness data**

The values of Vickers microhardness number data using Shapiro-Wilk test of artificial saliva group and the experimental groups were normally distributed as there was no significant difference among all groups (p˃0.05) (Table 5).

**Table 5: Normality of Vickers microhardness number (kg / mm2).**

| Groups | Baseline | | | Ph-cycling | | |
| --- | --- | --- | --- | --- | --- | --- |
|  | Statistic | df | P value | Statistic | df | P value |
| A.S | 0.889 | 10 | 0.163 | 0.943 | 10 | 0.585 |
| Theobromine | 0.963 | 10 | 0.816 | 0.927 | 10 | 0.418 |
| BAG | 0.939 | 10 | 0.540 | 0.966 | 10 | 0.848 |
| Nano-HAP | 0.928 | 10 | 0.426 | 0.963 | 10 | 0.824 |

1. Amaechi BT. Protocols to study dental caries in vitro: pH cycling models. Odontogenesis: methods and protocols. 2019:379-92.
